# Subtrochanteric femoral shortening osteotomy combined with cementless total hip replacement for Crowe type IV developmental dysplasia: a retrospective study

**DOI:** 10.1007/s10195-017-0466-7

**Published:** 2017-07-24

**Authors:** Giuseppe Rollo, Giuseppe Solarino, Giovanni Vicenti, Girolamo Picca, Massimiliano Carrozzo, Biagio Moretti

**Affiliations:** 1 Orthopedics and Traumatology Department, Vito Fazzi Hospital, Piazzetta Muratore, 73100 Lecce, Italy; 2 Department of Neuroscience and Organs of Sense, Orthopedics Section, Faculty of Medicine and Surgery Policlinico di Bari, University of Bari, Piazza Giulio Cesare 11, 70124 Bari, Italy

**Keywords:** Hip dysplasia, Hip replacement, Shortening osteotomy

## Abstract

**Background:**

Total hip replacement for high dislocation of the hip presents some difficulties, considering patients’ young ages, the abnormal hip anatomy and the high rate of complications. In this study, we present our experience in terms of clinical and radiological results in the treatment of Crowe type IV hips with subtrochanteric femoral shortening osteotomy and cementless total hip replacement.

**Materials and Methods:**

We retrospectively reviewed 15 patients with Crowe type IV hip dysplasia (two bilateral cases for a total of 17 hips) treated with cementless total hip replacement associated with shortening subtrochanteric osteotomies (nine transversal and eight Z-shape osteotomies) between March 2000 to February 2006. The mean follow-up was 88 months (range 63–133). Harris hip score, leg length discrepancy, neurological status, union status of the osteotomy and the component stability were the criteria of the evaluation. All complications were noted.

**Results:**

The mean HHS improved from 38.3 (range 32–52) to 85.6 (range 69–90). The mean preoperative leg length discrepancy was of 45 mm (range 38–70) and reduced to a mean of 12 mm (range 9–1.6) postoperatively. All osteotomies resulted healed at an average of 12.3 weeks (range 10–15). No cases of delayed union or nonunion were detected. Two patients (11%) showed early symptoms of sciatic nerve palsy which resolved uneventfully in 6 months. There was no migrations and none of the implants required revision.

**Conclusions:**

Cementless THA with shortening subtrochanteric osteotomy is an effective method in the treatment of patients with Crowe type IV development dysplasia of the hip.

**Level of evidence:**

IV.

## Introduction

Developmental dysplasia of the hip (DDH) is a common cause of secondary osteoarthritis of the hip in young adults. Abnormal contact stresses in the dysplastic hip predispose patients to develop earlier arthritic changes [1].

Total hip replacement (THR) is one of the treatment options and represents the standard of care for this condition, characterized by a wide range of deformities, from simple acetabular dysplasia to high dislocation of the hip [2]. THR in DDH is a technically challenging procedure for the orthopedic surgeon; different issues must be considered as patient’s young age and abnormal anatomy [3].

Patients affected by high DDH have a unique and proper anatomy; both acetabulum and proximal femur are often associated with well-defined patterns. Acetabulum is characterized by deficiencies in the anterolateral and superior zone making it difficult to obtain sufficient bony coverage of the cup. The proximal femur is hypo plastic with a narrow intramedullary canal and increased anteversion. Moreover, the muscles and soft tissues around the hip are shortened and the capsule is thickened [4].

The abductors insufficiency leads to a limp or to a frank Trendelenburg gait. The sciatic nerve is shortened predisposing to injury in case of limb lengthening greater than 4 cm; sciatic nerve palsy is reported from 0.8 to 13% for patients with hip dysplasia treated with hip replacement [5, 6].

To overcame the contractures and to reduce the hip without stretching the sciatic nerve, femoral shortening has been advocated as an adjunct to THR. Cementless THR with a subtrochanteric femoral shortening osteotomy, rather than a proximal shortening osteotomy with distal advancement of the great trochanter to restore abductor muscle function, has been described as it avoids the risk of fibrous non-union of the great trochanter [7].

In this retrospective study, we present our experience with subtrochanteric shortening osteotomy of the femur and implantation of cementless THR in patients with Crowe type IV DDH. The rationale for the use of cementless THR was to avoid introducing cement at the osteotomy site, which might interfere with healing.

## Materials and methods

We retrospectively analyzed charts, radiological and operative data of 15 patients (17 hips) with Crowe type IV DDH treated in our institution with primary cementless THR along with a subtrochanteric shortening osteotomy from March 2000 to February 2006. There were ten women and five men with a mean age of 38.6 years (range 28–68). Informed consent was obtained from all individual participants included in the study.

Inclusion criteria were the presence of severe hip pain and considerable difficulties in walking and performing daily activities supported by a radiographic diagnosis of mono- or bilateral Crowe type IV hip dysplasia. Patients with clinical evidence of nerve dysfunction before the surgery were not included in the study. Two patients had bilateral high hip dislocation and 13 had high dislocation of one hip and normal development of the other. Before surgery, the mean length leg discrepancy was 45 mm (range 38–90). One patient had previous bilateral Schanz osteotomy. The mean follow-up was 88 months (range 63–133).

Our standard pre-operative protocol for patients affected by Crowe IV deformity consisted in clinical evaluation of the hips, spine, knees and musculoskeletal system. Special attention was paid to the status of the sciatic nerve. In this regard, electromyography study was constantly performed in all patients before surgery. Evaluation of pre-operative Harris Hip Score (HHS), Body Mass Index (BMI), X-rays and CT study of the pelvis were also performed. Hip pain was measured with a visual analogue scale (VAS). An accurate preoperative planning was done in all patients with particular attention to the bone stock and the level of the subtrochanteric osteotomy.

### Operative technique

A spinal block was preferred in ten cases; the remaining five patients underwent general anesthesia. The direct Hardinge lateral approach was used, with patients in lateral decubitus.

Some authors begin the preparation on the femoral side, but we prefer starting from the acetabulum.

After resection of the femoral head through the femoral neck, the joint capsule was totally removed to provide a wide view of the acetabulum. In all patients, the acetabulum was placed at its natural level, and the femur was then inferiorized.

We began reaming the true acetabulum with small reamers and gradually enlarged the recess to contain a cementless component. The true acetabulum was reamed through the medial wall until the medial periosteum was seen. The cup was then impacted and fixed with one screw in seven cases and two screws in the remaining ten hips for augmenting the primary stability. The Allofit Acetabular Cup (Sulzer^®^, Switzerland) was used in five cases, while in the other 12 hips a Bantam Cup (DePuySynthes^®^, Warsaw, Indiana) was implanted. We used a metal on poly coupling in 15 hips and a ceramic on ceramic in two hips. The average size of the cup was 42 mm (range 38–46). In 11, two and four hips, 22-, 28-, 32- mm femoral heads were respectively implanted. In every patient, more than 85% of the cup was covered by the host bone, and the use of graft was therefore not necessary.

**Fig. 1:**
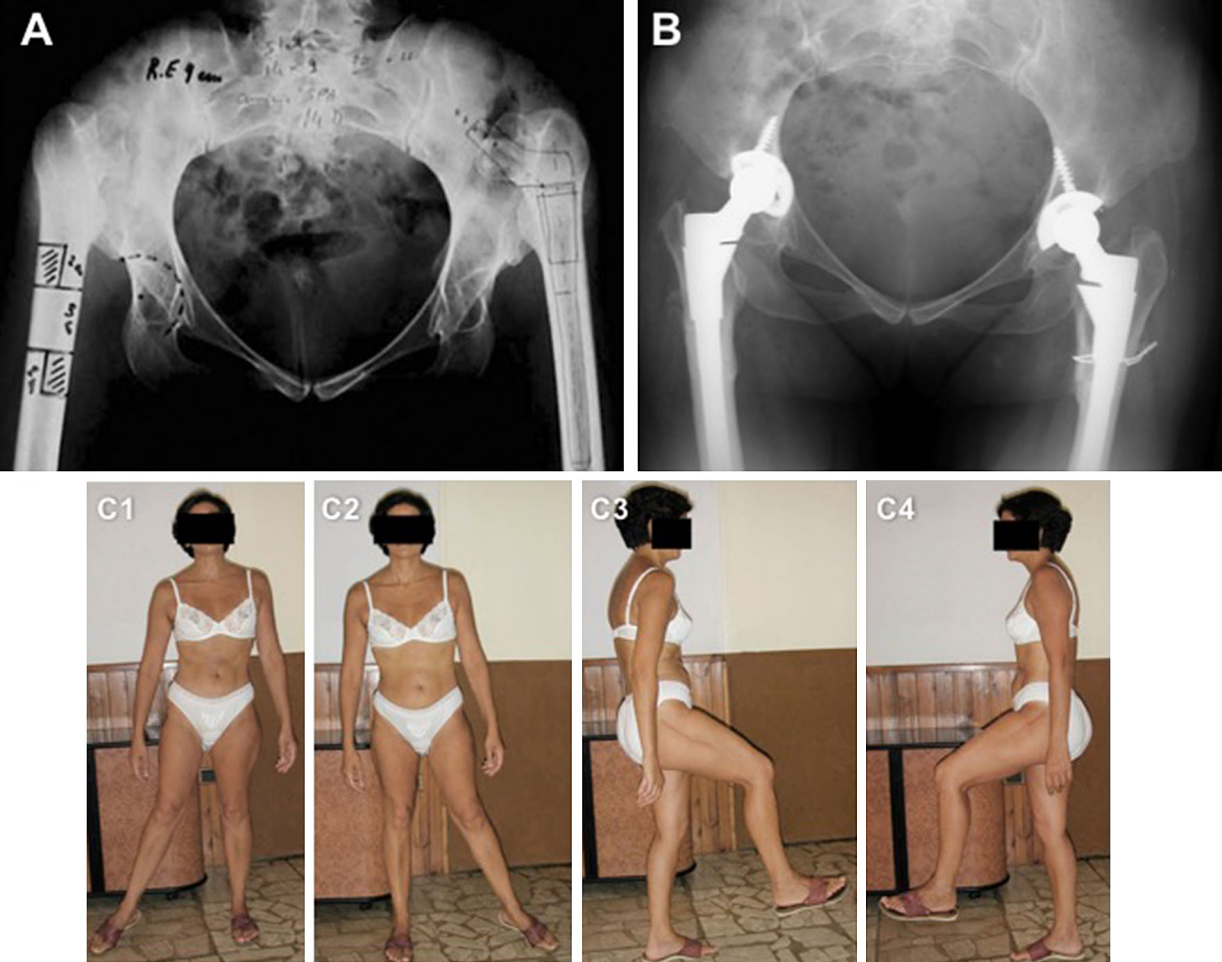
44-year-old female patient with bilateral DDH. **a** Pre-operative planning with the Z-shaped osteotomy. **b** X-ray at 2-year follow-up right hip surgery (1-year; *left* hip). Note the intra-op fracture on the *left* hip treated with an additional cerclage. **c1**–**4** Clinical images at 5-year follow-up right hip surgery (4-year; *left* hip)

**Fig. 2:**
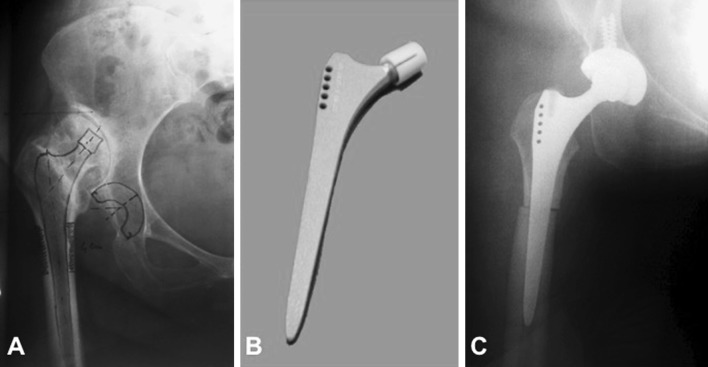
39-year-old male patient with unilateral Crowe type IV DDH. **a** Pre-operative planning with cementless THA and transverse shortening osteotomy. **b** Quadrangular cross-section CSR Japan stem (Sulzer^®^, Switzerland). **c** Post-operative X-ray

After the acetabular component was placed, the femoral medullar cavity was prepared with intramedullary reaming and broaching before executing the osteotomy. If a Schanz osteotomy had been performed previously (two hips), the shortening was combined with correction of the angulation from the previous osteotomy. In our series, nine hips received a transverse subtrochanteric shortening osteotomy; eight hips had a Z-shaped osteotomy (Figs. 1a, 2a, c). This latter osteotomy type consists of removing a similar amount of longitudinal half of the femoral bone, in the proximal and distal regions of the osteotomy. In our study, the proximal and distal steps were planned respectively on the lateral and medial sides. The proximal longitudinal half of the osteotomy was performed in the midline by holding the femur in a position with desired anteversion (10°–15°).

To determine the amount of shortening, the proximal femur implanted with the femoral trial component was reduced into the prepared true acetabulum. The extension of resection was decided depending on the pre-operative radiographic measurements and the intraoperative examination by subtracting the expected leg lengthening from the expected descent of the tip of the greater trochanter. Bookmarked levels on the distal fragment were osteotomized, and then the trial was inserted. In case of a transverse osteotomy, the rotational alignment of both the proximal fragment and the distal fragment was adjusted to allow approximately 10°–15° of anteversion of the femoral component and optimal rotational alignment of the proximal fragment relative to the distal one. Leg length, off-set and stability against dislocation were then tested. If these were acceptable, the osteotomy was provisionally stabilized with bone clamps. After having achieved stable fixation of the osteotomy, final preparation of the femur was undertaken, including repeat reaming and broaching until optimal cortical contact was achieved especially distal to the osteotomy site. Finally, the definitive femoral component was inserted. No osteotomies were grafted. The length of the resected femur was on average 39 mm (range 30–55). The type of osteotomy was chosen by pre-operative and intra-operative parameter evaluation. A Z-shaped osteotomy was preferred in patients with a good bone stock and a BMI lower than 28. Implants were chosen to guarantee primarily the rotational stability. In 12 hips the S-ROM Modular Hip System (DePuySynthes^®^, Warsaw, Indiana) was used while in five cases a CSR Japan stem was used (Sulzer^®^, Switzerland). The S-ROM system (Fig. 1b) has a proximally modular cementless stem, which achieves rotational stability in the distal femur through its splines; the distal portion has polished flutes that cut into the distal cortices of the femoral canal and further increase rotatory resistance [8]. The Japan CSR stem (Fig. 2b) is a Zweymüller type stem characterized by a rectangular cross-section, which improves implant rotational stability and stress forces distribution.

At the end of the surgery, the contracture of the adductor tendons was assessed as well as the motion in abduction, in order to evaluate the necessity of a percutaneous partial adductor tenotomy (performed in eight patients).

In all patients, Venous Thromboembolism Prophylaxis consisted of subcutaneous injection of 4000 UI/day of enoxaparin. Standard suction drains with an external diameter of 3 mm were used. These were attached by a closed system to prevacuumed glass bottles. The drains were removed in the second day post-op. Standard short-term antibiotic prophylaxis with cefazolin was applied.

Table 1 summarizes patients and implants details.

**Table 1:** Patients demographics and implants

Variables	Number
Patients	15
Male	10
Female	5
Hips	17
Right	7
Left	6
Bilateral	2
Mean age at time of surgery (years)	38.6 (28–68)
Mean follow-up time (months)	88 (63–133)
Osteotomy	
Z-shape	8
Transverse	9
Mean resected femur (mm)	39 (30–55)
Femoral stems	
S-ROM	12
CSR Japan	5
Acetabular components	
Bantam	12
Allofit	5

### Postoperative care

After surgery the leg was placed with the hip in full extension, and the knee flexed to reduce tension forces on the sciatic nerve, as reported by Morscher et al. [9].

We allowed partial weight bearing (15–20 kg) with crutches during the first 3 months postoperatively, with progression to full weight bearing the following weeks. During the hospital stay, the patients’ gait was supervised by a physiotherapist, but the patients had no specific training.

Patients were followed up clinically and radiographically at 1, 3, 6 and 12 months after surgery; then every year. Clinical examination was performed using the Harris Hip Score. Patients were observed for any limp and leg length discrepancy was measured from the anterior superior iliac spine to the medial malleolus as the patient lying supine. The residual hip pain using a visual analogue scale (VAS) was also determined. Radiographs of the hip were evaluated using the femoral component zones described by Gruen et al. 11. Attention was paid to the union status of the osteotomy site, any sign of loosening and implant subsidence. Osteolysis was defined as an appearance of a focal area of bone resorption evidenced by a cystic lesion. All complications were registered.

### Statistics

We used the *t* test, with 5% significance level, to check the difference in continues variables.

## Results

The mean follow-up was 88 months (range 63–133). No patients were lost at follow-up.

The average time of the surgery was 125 min (range 118–150).

The mean preoperative HHS was 38.3 (range 32–52) and significantly improved post-operatively to 85.6 (range 69–90) (*p* < 0.05) (Fig. 1c). The best results were found in pain and function scores, while the range of motion presented the lowest improvement.

The mean preoperative hip pain score on the VAS was 8.2 (range 7–9) and was significantly lower at the last follow-up 3.2 (range 2–4) (*p* < 0.05).

A Trendelenburg gait was present in all 15 patients before surgery and remained in two of them after surgery.

The mean preoperative leg length discrepancy was of 45 mm (range 38–70) and reduced to a mean of 12 mm (range 9–1.6) postoperatively.

One (6%) intraoperative fracture of the medial femoral cortex occurred during the implantation of an S-ROM stem in a patient with bilateral DDH. The fracture required an additional cerclage (Fig. 2b). However, no progression into varus deformity occurred, and stable fixation of the stem was eventually achieved.

Two patients (11%) showed early symptoms of sciatic palsy. They had a significant swelling in the buttock, as well as signs of sciatic nerve irritation (paresthesia in the ipsilateral foot). None of them reported any previous surgery which could have been considered as predisposing factors.

After 3 months, nerve palsy was still present in one patient. The electromyography study was then performed, showing diffuse slowing of the nerve conduction velocity both in sensitive and motors fibers. At 6 months postoperatively the two patients had a complete resolution of symptoms and recovery of sciatic nerve function.

Radiographic analysis showed that three (18%) femoral stems had radiolucent lines less then 2 mm in zones 1, 2–3 and 7, respectively. However, in these three cases no definite radiographic or clinical indication of implant loosening was observed. We did not observe any radiolucent lines or osteolityic lesions suggestive of loosening in any of the acetabular components. There was no migration of every implant and none of them required revision. Healing of the osteotomy site was assessed with radiological union being defined as cortical continuity on both the AP and the lateral views and ability to walk without pain. All osteotomies that resulted healed at an average of 12.3 weeks (range 10–15). There was no significant difference in time to union between different type of osteotomies (*p* < 0.05). No infection or deep venous thrombosis were detected.

The results are summarized in Table 2.

**Table 2:** Patients clinical results

Variables	Pre-operative	Post-operative
Harris Hip Score	38.3 (32–52)	85.6 (69–90)
Hip pain on the VAS	8.2 (7–9)	3.2 (2–4)
Leg length discrepancy (mm)	45 (38–70)	12 (9–1.6)
Trendelenburg gait (*n*)	15 pts.	2 pts.

## Discussion

Patients with high DDH can be successfully treated with cementless THR. Nevertheless, this procedure is technically demanding and associated with high complications rates. The first problem to deal with patients with Crowe type IV DDH is about the position of the center of rotation of the new cup. Although some good results have been reported placing the cup in the new acetabulum [10, 11], the native acetabulum represents the best choice in terms of biomechanical concepts [12, 13]. In following this principle Nagoya et al. [7] observed no loosening of the acetabular component during a mean follow-up of 8.1 years (range 4–11.5). In our series, the acetabular cup was always placed in the native socket. No grafts were used, and the cup was always fixed using one or two screws for augmenting the primary stability. No case of cup loosening was reported at a mean follow-up of 88 months (range 63–133).

When the acetabular cup is placed in the true acetabulum, reduction of the hip is nearly impossible due to the shortened soft tissues. This can be addressed by two different techniques. A one-step surgery combining THA with a shortening osteotomy represents the standard approach for patients with high DDH. On the contrary, some authors [6, 14] recommend a two-step surgery: the first step, dedicated to the release of peri-joint tissues (progressive distraction of the femur), can minimize the risk of postoperative nerve palsy secondary to leg lengthening, avoiding femoral osteotomy and favoring the next surgery of joint replacement. Progressive distraction of the femur is generally obtained through positioning of external fixators and hip reduction is obtained without sacrificing femoral length. The main advantage of the two-step technique is to prevent early dislocations. The distraction time determines soft tissue lengthening and allows reduction of forces around the prosthetic implant. However, we strongly believe that the same outcomes can be achieved in one-step surgery technique when careful and precise soft tissue release is obtained, as confirmed in our series.

Femoral shortening can be achieved by proximal or distal osteotomies. When a proximal femoral osteotomy is used, the femoral metaphysis is resected. Moreover, trochanteric non-union is a potential risk of this procedure [13, 15]. In our opinion subtrochanteric shortening osteotomy represents a safer option, generating better results in terms of union. Additionally, with this technique the femoral metaphysis is preserved for future revision.

Subtrochanteric transverse osteotomy is the most common type used by the several authors [16–19]; it allows intra-operative measurement and excision of the over-lapping segment, with technically results easier to perform, and is associated with good results, relatively quick healing time, and low rates of non-union and malunion. Limited bone contact area represents a major disadvantage of transverse osteotomy, which may interfere with bone healing process.

Double-Chevron subtrochanteric osteotomy was primarily described by Becker and Gustilo [20] in 1995 and later adopted and eventually modified by other authors [21]. Double-chevron complex osteotomy techniques provide specific contact geometry and enhanced torsional stability; however, it remains a demanding procedure not always possible, especially in patients with low bone stock.

Oblique osteotomy technique was used by Dallari et al. [22] in 19 cases and one non-union occurred (94.73% complete healing).

Z-shaped subtrochanteric osteotomy is largely described by authors [23, 24]. This osteotomy provides larger bone contact surface and greater stability, avoiding torsional stress along the femoral stem. However, it is not always simple to perform, especially in patients with low bone stock; furthermore, the introduction of modular stem prosthesis with metaphyseal sleeve helps to reduce the amount of rotational stresses also when lower demanding osteotomies are performed.

Recently Muratli et al. [25], compared biomechanical outcomes of four osteotomy techniques on cadaveric specimens: transverse, oblique, Z-subtrochanteric and double Chevron. Authors concluded that intramedullary stability was linked primarily to the cross-sectional geometry of the femoral components, and its distal rotational stability reduced the role of the type of osteotomy, allogeneic strut graft and cerclages. We strongly believe that Z-shape osteotomy provides superior correction of antetorsion and angulation and should be performed whenever possible. In particular, we recommend the use of one-step surgery with femoral Z-shaped shortening osteotomy for patients with good bone stock and BMI lower than 28. Transverse osteotomy is an available option in the remaining cases. Both of them guarantee a good abductor strength and a good range of motion.

Common complications in femoral shortening osteotomy are fracture and non-union or delay at the osteotomy site. Intraoperative fracture during insertion of the femoral component has been reported to range from 5 to 22% [19, 26]. We identified only one intraoperative fracture (6%) of the medial femoral cortex occurring during the implantation of an S-ROM stem requiring additional cerclage. However, no progression into varus deformity occurred, and stable fixation of the stem was eventually achieved. In our series, all the resulting osteotomies healed at an average of 12 weeks (range 10–15). There was no significant difference in time to union between different types of osteotomies. This results are broadly in line with those presented in other studies using our same osteotomy techniques [7, 19, 23].

Nerve palsy complications are one of the biggest issues when dealing with patients affected by DDH. Sciatic nerve palsy is reported from 0.8 to 13% for patients with hip dysplasia treated with hip replacement [5, 6, 24]. Leg lengthening more than 4 cm was suggested to be an indication for femoral shortening osteotomy to prevent nerve palsy. However, Eggli et al. [27] found no relationship between leg lengthening and nerve palsy in 508 consecutive total hip replacements. Otherwise they statistically correlated the amount of difficulty of surgery to the occurrence of nerve palsy. In our series, two patients (11%) showed early symptoms of sciatic palsy. None of them reported any previous surgery, which could have been considered as predisposing factors. However, both patients had a complete resolution of symptoms and recovery of the sciatic nerve function at 6 months postoperatively.

Cementless stems have been used in DDH in the hope that better results could be obtained in young patients by biological fixation. Cementless stems can also be used in cases of narrow femoral canals, where the use of a cemented stem would lead to a cement mantle of inadequate thickness [28]. Moreover, these devices avoid introducing cement at the osteotomy site, which might interfere with healing. Lai et al. [6] reported on 56 Crowe type IV hips treated with a cementless total hip replacement. At an average follow-up of 12.3 years, there were no stem revisions. Yalcin et al. [29] observed no symptomatic loosening in their series of cementless THA combined with shortening transverse osteotomy for severely dysplastic hips with a mean follow-up of 62 months. In our series, radiographic analysis showed that three femoral stems had radiolucent lines less then 2 mm. However, in these three cases no definite radiographic or clinical indication of implant loosening was observed. There was no migration of every implant and none of the implants required revision.

In conclusion, subtrochanteric shortening osteotomy combined with cementless total hip replacement is an effective and reliable method for treatment of Crowe type IV development dysplasia. This procedure allows the restoration of a more normal limb length with good clinical results and a low rate of neurological complications.
